# Enhanced Delivery of Nucleic Acids to Insect Cells by Star Polycation Formulation

**DOI:** 10.3390/insects17080869

**Published:** 2026-08-20

**Authors:** Niayesh Shahmohammadi, Taegeun Song, Falguni Khan, Sima Majidiani, Yonggyun Kim

**Affiliations:** School of Smart-Green, College of Life Sciences & Engineering, Gyeongkuk National University, Andong 36279, Republic of Korea; niayeshshahmohammadi@gmail.com (N.S.); xorms5095@naver.com (T.S.); falgunikhan2942@gmail.com (F.K.); s.majidianii@gmail.com (S.M.)

**Keywords:** RNAi, formulation, delivery, Lepidoptera, thrips, aphid

## Abstract

Gene delivery to target cells is required for bioengineering or medical/agricultural applications. However, the hydrophobicity of the cell membrane always makes it resistant to polar nucleic acids. This physicochemical barrier is usually overcome by a nano-formulation to hide the polarity. This study evaluated a specific formulation called star polycation (SPc) regarding its efficacy in DNA/RNA delivery to insect cells. The delivery efficiency of the SPc formulation was assessed by transient expression of *green fluorescence protein* (*GFP*) in Sf9 cells, in which SPc formulation significantly enhanced the gene expression compared with an unformulated vector. In vivo transient expression (IVTE) was performed by injection of the expression construct with the SPc formulation into larvae of *S. exigua*. Fluorescence was detected in all four tissues, namely, epidermis, midgut, hemocyte, and fat body, where the SPc formulation enhanced the expression in most tissues except epidermis. Under this IVTE condition, an additional injection of SPc-formulated dsRNA specific to *GFP* suppressed the gene expression significantly more than the unformulated vector. The enhanced RNA interference (RNAi) efficiency caused by the SPc formulation was confirmed against four endogenous genes of *S. exigua*, namely, *Snf7*, *PSMB5*, *vATPase*, and *α-tubulin*, by either injection or feeding of dsRNA. These RNAi treatments were lethal to *S. exigua*, in which dsRNA specific to *vATPase* formulated with SPc resulted in almost 80% mortality through oral delivery. A similar oral toxicity by SPc-formulated dsRNA was demonstrated in another lepidopteran *Plutella xylostella*. The oral delivery of dsRNA was applied to control sucking insects such as aphids and thrips by spraying SPc-formulated dsRNA onto plant surfaces. The sprayed dsRNA labeled with Cy3 fluorescence was detected in the internal tissues of plant leaves, in which the penetration of dsRNA into the plant tissues was further accelerated by SPc formulation. The SPc formulation of dsRNA specific to *vATPase* was effective at killing the sucking insects by spraying on plant surfaces. These results suggest the application of an SPc formulation to deliver DNA/RNA to insect cells.

## Simple Summary

RNA interference (RNAi) is a promising approach for controlling insect pests because it can selectively silence essential genes. However, its practical use is often limited by the poor stability and cellular uptake of double-stranded RNA (dsRNA). In this study, we evaluated a star polycation (SPc) nanoparticle as a carrier for delivering DNA and dsRNA to insect cells. The SPc formulation improved the delivery and expression of an *EGFP* reporter plasmid in insect cells and in several tissues of the beet armyworm *Spodoptera exigua*. It also enhanced dsRNA-mediated gene silencing and increased insect mortality following injection, oral feeding, or foliar application. Similar improvements were observed in the diamondback moth, green peach aphid, and western flower thrips. SPc also promoted the penetration of sprayed dsRNA into hot pepper leaves. These findings demonstrate that SPc can improve nucleic acid delivery and RNAi efficiency in different insect pests and may provide a useful platform for developing sprayable RNA-based pest control products.

## 1. Introduction

RNA interference (RNAi) is a cellular process in eukaryotes to control gene expression at the posttranscriptional level [1]. It operates in at least three different pathways: PIWI-interacting RNA (piRNA), microRNA (miRNA), and small interfering RNA (siRNA). The piRNA pathway uses PIWI to mature piRNA precursors and guides the recognition and silences transposon-derived RNAs [2]. The miRNA pathway endogenously regulates target gene expression based on genome-encoded miRNA precursors through an RNA-induced silencing complex [3]. The siRNA pathway interferes with gene expressions using exogenous (or synthetic) double-stranded RNA (dsRNA) and is widely exploited for bioengineering or medical/agricultural purposes through nanocarriers [4].

Pest control in agricultural crops heavily depends on chemical pesticides and gives rise to serious toxicological issues in humans and ecosystems [5]. Alternative control tactics are now urgent, in which RNAi has been considered to be an optimal option because of its high specificity to target pests [6,7]. This possibility was first demonstrated by a transgenic corn expressing dsRNA specific to *Snf7* of corn rootworm *Diabrotica virgifera virgifera* [8], followed by commercialization of the transgenic corn. Then, movement of the practical use of dsRNA was driven by a spray-induced gene silencing (SIGS) strategy and led to the development and commercialization of Ledprona consisting of sprayable dsRNA targeting Colorado potato beetle *Leptinotarsa decemlineata* in 2023 in accordance with US Environmental Protection Agency (EPA) registration [9,10]. SIGS-based dsRNA application can be expanded to control other pests.

Although dsRNA delivery is crucial for effective RNAi to control pests, several factors limit the efficacy of dsRNA applications to control various insect pests. After oral intake, the dsRNA in the gut lumen or extracellular matrix may be vulnerable to the attack of dsRNases [11]. Moreover, RNAi specific to these dsRNase genes significantly enhanced the RNAi efficacy in insects and nematodes [12,13]. Another barrier would be the cellular uptake of dsRNA to the target cells, in which dsRNA can get access to the cells via a direct passage through channel protein using SID-like or receptor-mediated endocytosis [14]. The uptake pathway and its efficiency are highly variable between different tissues [15]. Moreover, the internalized dsRNA can be degraded by forming an endosome to be fused with lysosome, especially in lepidopteran insects [16].

Nanoparticle-based formulations are useful tools for delivering dsRNA, where they include clay, chitosan, liposome, carbon, gold, and silica to shield dsRNA molecules from enzymatic degradation and facilitate translocation across cell membranes [17]. Among the formulations, the star polycation (SPc) nanoparticle has four peripheral amino acid functionalized arms and positively charged outer shells to bind the negatively charged dsRNA. The SPc–dsRNA complex can decrease the zeta potential to +4.0 mV while the size increases during formulation [18]. SPc was designed and synthesized for transdermal dsRNA delivery system in plants to control plant diseases and sucking insects [18,19]. Thus, SPc nano-formulation is highly likely to be an optimal tool to deliver dsRNA for pest management, but it needs further analysis regarding the delivery of various nucleic acids in RNAi-resistant lepidopteran hosts. The transdermal movement of dsRNA formulated in SPc needs to be assessed regarding insecticidal activity against sucking insects such as thrips and aphids. This study evaluated the delivery of the dsRNA/DNA construct in an SPc formulation and subsequent gene expression or RNAi efficiency.

## 2. Materials and Methods

### 2.1. Insect Rearing

Larvae of *Spodoptera exigua* were originally collected from Welsh onion fields in Andong, Korea, and maintained in the laboratory at 25 ± 2 °C and 70 ± 5% relative humidity (RH) with a 16:8 h (light:dark) photoperiod. The larvae were reared on fresh cabbage leaves and progressed through five distinct larval instars (L1–L5). Larvae of *Plutella xylostella* were also reared on cabbage leaves under the same environmental conditions and underwent four instars (L1–L4). Adults were provided with a 10% sucrose solution for oviposition.

The laboratory colony of *Frankliniella occidentalis* was established by individuals collected from hot pepper fields in Andong, Korea. Both larvae and adults were maintained under the same environmental conditions described above and reared on germinated kidney beans (*Phaseolus coccineus* L. cv. Kangnam). Newly hatched larvae were transferred daily to round breeding dishes (100 mm diameter × 40 mm height; SPL Life Sciences, Pocheon, Republic of Korea).

Similarly, laboratory colonies of *Myzus persicae* were established from field populations collected from *Capsicum annuum* in greenhouses located in Andong, Korea. The aphid colonies (consisting of nymphs and adults) were subsequently maintained on broccoli (*Brassica oleracea* var. italica) under controlled conditions (25  ±  2 °C, 70  ±  5% RH, 14:10 h light:dark photoperiod).

### 2.2. Chemicals

Pentaerythritol (98%) was purchased from Alfa Aesar (Ward Hill, MA, USA). 2-Bromo-2-methylpropionyl bromide, triethylamine (TEA), tetrahydrofuran (THF, anhydrous), 2-(Dimethylamino)ethyl methacrylate (DMAEMA, 99%), copper(I) bromide (CuBr, 99.999%), phosphotungstic acid, and N,N,N′,N″,N″-pentamethyldiethylenetriamine (PMDETA) were purchased from Sigma-Aldrich Korea (Seoul, Republic of Korea).

### 2.3. RNA Extraction and cDNA Synthesis

Total RNA was extracted from one *S. exigua* larva, approximately 20 aphids (*M. persicae*), or approximately 50 thrips (*F. occidentalis*) per biological replicate using TRIzol reagent (Invitrogen, Carlsbad, CA, USA) according to the manufacturer’s instructions. The extracted RNA was resuspended in nuclease-free water, and its concentration was determined using a NanoDrop spectrophotometer (Thermo Scientific, Wilmington, DE, USA). First-strand cDNA was synthesized from 100 ng of total RNA using RT Premix (Intron Biotechnology, Seoul, Republic of Korea), which contains an oligo(dT) primer, according to the manufacturer’s protocol.

### 2.4. RT-PCR and RT-qPCR

Synthesized cDNAs were used as templates for polymerase chain reaction (PCR) using Taq DNA polymerase (GeneALL, Seoul, Republic of Korea) and gene-specific primers (Table S1). PCR amplification was performed under the following conditions: initial denaturation at 95 °C for 3 min, followed by 35 cycles of denaturation at 95 °C for 1 min, annealing at 55 °C for 1 min, and extension at 72 °C for 1 min. Each 25 μL PCR reaction contained 1 μL of cDNA template, 1 μL of dNTP mixture (2.5 mM each), 1 μL each of forward and reverse primers (10 pmol), 1 μL of Taq DNA polymerase (2.5 U μL^−1^), and nuclease-free water to the final volume. Quantitative real-time PCR (RT-qPCR) was performed using a StepOnePlus Real-Time PCR System (Applied Biosystems, Singapore) with Power SYBR Green PCR Master Mix (Applied Biosystems, Carlsbad, CA, USA) according to the guidelines of Bustin et al. [20]. Each 20 μL reaction contained 10 μL of Power SYBR Green PCR Master Mix, 2 μL of cDNA template (60 ng μL^−1^), 1 μL each of forward and reverse primers, and nuclease-free water to the final volume. Ribosomal protein L32 genes for *S. exigua* (*Se-RL32*) and *P. xylostella* (*Px-RL32*), as well as elongation factor 1α genes for *M. persicae* (*Mp-EF1α*) and *F. occidentalis* (*Fo-EF1α*), were used as reference genes (Table S1). Melting curve analysis was performed to confirm the amplification specificity. Relative gene expression was calculated using the comparative Ct (2^−ΔΔCt^) method with three independent biological replicates.

### 2.5. dsRNA Synthesis and Cy3 Fluorescence Labeling

Gene-specific fragments were amplified by PCR using primers containing the T7 promoter sequence at their 5′ ends (Table S1). The resulting PCR products were used as templates for in vitro transcription with the MEGAscript RNAi Kit (Ambion, Austin, TX, USA) according to the manufacturer’s instructions. The synthesized dsRNAs were mixed with Metafectene PRO at a 1:1 (*v*/*v*) ratio and incubated at room temperature for 30 min to allow the formation of dsRNA–liposome complexes. RNAi efficiency was subsequently evaluated by RT-qPCR. As a negative control (dsCON), a 520 bp fragment of *green fluorescent protein* (*GFP*) gene was amplified from pUB-GFP plasmid (Plasmid #11155, Addgen, MA, USA) using a specific primer pair (Table S1).

Cy3 labeling of dsRNA was performed using Cy3 Label IT Nucleic Acid Labeling Kit (Mirus Bio, Madison, WI, USA) under low-light conditions to minimize photobleaching. Briefly, 5 μg of dsRNA was mixed with 1× labeling buffer and reconstituted Cy3 labeling reagent in a final reaction volume of 50 μL prepared with nuclease-free water. The reaction mixture was gently mixed, briefly centrifuged, and incubated according to the manufacturer’s instructions to allow covalent attachment of Cy3 to the dsRNA. Excess unbound dye was removed using the purification procedure supplied with the kit.

For the Cy3 confocal analysis, Cy3-labeled dsRNA and DAPI-stained nuclei were imaged separately using a confocal microscope (Zeiss Zen 3.4, Zeiss, Oberkochen, Germany). Cy3 fluorescence was detected using the FITC channel and was displayed in green, whereas DAPI was displayed in blue.

### 2.6. Preparation of Recombinant pIB-EGFP Vector

The full-length open reading frame (ORF; 738 bp) of *enhanced green fluorescent protein* (*EGFP*) was amplified from a recombinant Autographa californica multiple nucleopolyhedrovirus (AcMNPV) expressing *EGFP* [21]. The amplified EGFP ORF was cloned into the pIB/V5-His TOPO vector (Invitrogen, Carlsbad, CA, USA) for insect in vivo transient expression (IVTE). Recombinant plasmids were screened by PCR, and the orientation and sequence of the inserted EGFP ORF were confirmed by DNA sequencing to verify its correct reading frame downstream from the baculoviral OpIE2 promoter.

### 2.7. Preparation of SPc and Nano-Formulation

SPc was synthesized by atom transfer radical polymerization (ATRP), as previously described [22] with minor modifications. Briefly, pentaerythritol (98%, Alfa Aesar) was recrystallized from water and dried under vacuum before use. The four-arm ATRP initiator (Pt–Br) was prepared by the dropwise addition of 2-bromo-2-methylpropionyl bromide (253 mg, 1.11 mmol) to a solution of pentaerythritol (25 mg, 0.18 mmol) and triethylamine (TEA, 111.3 mg, 1.11 mmol) in anhydrous tetrahydrofuran (THF, 20 mL) at 0 °C. The reaction mixture was stirred at room temperature for 24 h, quenched with methanol, and the solvent was removed under reduced pressure. The residue was recrystallized from cold diethyl ether to obtain Pt–Br as a white powder (50 mg, 40% yield). The structure of Pt–Br was confirmed by ^1^H NMR (400 MHz, CDCl_3_): δ 4.33 (s, 8H) and 1.94 (s, 24H).

For SPc synthesis, 2-(dimethylamino)ethyl methacrylate (DMAEMA, 99%) was distilled under vacuum prior to use. Pt–Br (40 mg, 0.055 mmol), DMAEMA (2.2 g, 7.7 mmol), and anhydrous THF (8 mL) were added to a reaction flask and degassed with nitrogen for 30 min. CuBr (46 mg, 0.22 mmol) and N,N,N′,N″,N″-pentamethyldiethylenetriamine (PMDETA, 110 mg, 0.44 mmol) were then added, and ATRP was carried out at 60 °C for 7 h under a nitrogen atmosphere. After polymerization, THF was removed using a rotary evaporator, and the crude polymer was purified by dialysis against deionized water (four changes), followed by lyophilization (or drying under reduced pressure) to obtain SPc as a white powder (1.8 g, 80% yield).

For the nano-formulation, 0.1 g of SPc powder was dissolved in 10 mL of distilled water and sonicated for 30 min. The SPc suspension was then mixed with dsRNA or pIB-EGFP at different mass ratios, followed by incubation at room temperature for 1 h to allow nanoparticle formation.

### 2.8. Characterization of SPc Nanoparticles by Transmission Electron Microscopy

The morphologies of SPc-dsRNA and SPc-pIB-EGFP nanoparticles were examined by transmission electron microscopy (TEM). Briefly, a drop of each nanoparticle suspension was placed onto a carbon-coated copper grid (Electron Microscopy Sciences, Morgantown, PA, USA) and negatively stained with 2% phosphotungstic acid (Sigma-Aldrich, Republic of Korea). After incubation at room temperature for 10 min, excess stain was removed using filter paper, and the samples were air-dried. The nanoparticles were observed using a transmission electron microscope (H-7650, Hitachi, Tokyo, Japan), and representative images were captured.

### 2.9. Sf9 Cell Culture and pIB-EGFP Treatment

A *Spodoptera frugiperda* cell line (Sf9; IPLB-Sf21-AE) was maintained in 25 cm^2^ tissue culture flasks (Nunc, Roskilde, Denmark) at 28 °C in TC-100 insect cell culture medium supplemented with 10% fetal bovine serum (FBS; Welgene, Daegu, Republic of Korea) and 1% antibiotic-antimycotic solution (100×; Gibco, New York, NY, USA). For the pIB-EGFP delivery assay, Sf9 cells were seeded in 6-well plates at a density of 2 × 10^6^ cells per well in 2 mL of TC-100 medium and incubated for 24 h at 28 °C. The plasmid was purified and mixed with the transfection agent Metafectene PRO (Biontex, Planegg, Germany) at a 1:1 (*v*/*v*) ratio and incubated at 25 °C for 30 min to facilitate liposome formation. Cells were then treated with 1 µg of pIB-EGFP per well, either alone or formulated with SPc at a pIB-EGFP:SPc mass ratio of 1:30. Following treatment, the cells were incubated at 28 °C and examined at 6, 12, 24, and 48 h. EGFP expression was evaluated by fluorescence microscopy, and relative *EGFP* transcript levels were quantified by RT-qPCR as described above. Each treatment was independently replicated three times (*n* = 3), with one independently treated Sf9 cell culture serving as one biological replicate.

### 2.10. Delivery of dsRNA/pIB-EGFP and Fluorescence Detection

Deliveries of dsRNA and pIB-EGFP were performed by either microinjection or oral feeding depending on the insect species. For microinjection, fifth instar (L5) *S. exigua* larvae were injected with 1 μL of dsRNA (200 ng) or pIB-EGFP (500 ng) into the proleg using a microsyringe (Hamilton, Reno, NV, USA).

For oral delivery, last-instar *S. exigua* and *P. xylostella* larvae, as well as *F. occidentalis* larvae, were used. Prior to feeding, the insects were starved for 1–3 h to promote rapid ingestion of the treated diet. Fresh cabbage leaf pieces (2 × 2 cm square) were used for *S. exigua* and *P. xylostella*, whereas germinated kidney beans were used for *F. occidentalis*. The diets were immersed in dsRNA or pIB-EGFP suspensions for 5 min, while control diets were treated with dsCON. After treatment, the diets were placed on filter paper for approximately 10 min to remove excess moisture and were then provided to the insects.

Oral delivery of dsRNA and pIB-EGFP in *M. persicae* was performed using a parafilm-based feeding chamber prepared in sterile Petri dishes. A feeding solution containing 30% (*w*/*v*) sucrose and dsRNA or pIB-EGFP at the concentrations described above was applied to the inner surface of a stretched parafilm membrane. The membrane was gently punctured at the feeding area to facilitate stylet penetration. Adult aphids were placed on the outer surface of the membrane and maintained at 25 ± 1 °C and 70 ± 5% relative humidity with a 14:10 h light photoperiod to allow ingestion of the treated diet.

Following treatment, insects were dissected under sterile conditions, and the dissected tissues were mounted on glass slides using 4% paraformaldehyde. Fluorescence-positive cells were observed using a Leica DM2500 fluorescence microscope (Leica Microsystems, Wetzlar, Germany) at 200× magnification. Fluorescence intensity was quantified using ImageJ software (v1.54, National Institutes of Health, Bethesda, MD, USA), and each experiment was performed with three biological replicates.

### 2.11. Insecticidal Bioassay of dsRNA

To assess dsRNA insecticidal activity, a feeding assay was conducted using kidney beans for thrips. The germinated bean seed kernels were soaked in either naked or nanoparticle-formulated dsRNA for 5 min and then air-dried for 10 min under sterile conditions. The treated kernels were placed in the breeding dishes, and L1/L2 *F. occidentalis* larvae were allowed to feed on these kernels for 12 h. Afterwards, dsRNA-treated kernels were replaced with fresh, untreated kernels and continued for three days. Each treatment was replicated three times. Mortality was recorded at 3 days post-feeding.

To deliver dsRNA orally for aphids, a parafilm-based feeding chamber was prepared in a sterile Petri dish. A 100 µL droplet of 30% (*w*/*v*) sucrose solution mixed with dsRNA (naked or nanoparticle-formulated) was placed on the inner side of a stretched parafilm layer. A small puncture was made at the droplet site to allow aphid access. Adult aphids (10 per replication) were placed on the outer surface of the parafilm and allowed to feed for 72 h under controlled conditions (25 ± 1 °C, 70 ± 5% RH, 14:10 h light:dark). Each treatment was replicated three times.

For oral delivery to lepidopteran larvae, fresh cabbage leaves (2 × 2 cm square per replicate) were immersed in naked or SPc-formulated dsRNA solution (500 µg/mL) for 5 min and air-dried for approximately 10 min. The treated leaves were then provided to 10 larvae of *S. exigua* or *P. xylostella* per replicate. Each treatment was independently replicated three times (*n* = 3). Mortality was recorded at 3 days post-feeding.

### 2.12. Control Efficacy of dsRNA in Hot Pepper Pot

Each pot (12 cm diameter × 12 cm height) contained one hot pepper plant approximately 15 cm in height. The pots were arranged in four rows, with 40 cm between rows and 50 cm between adjacent pots within each row. Before dsRNA application, each plant was infested with 40–60 adult aphids or L1/L2 thrips larvae. The pots were arranged in a randomized complete block design, with three blocks serving as biological replicates. Each plant was sprayed with 5 mL of dsRNA solution at a concentration of 250 ppm. Control plants were treated with an equal volume of dsCON solution at the same concentration. For each replicate, relative survival was calculated as the number of insects at each sampling time divided by the corresponding initial number at 0 DAT × 100. Control efficacy was then calculated relative to the dsCON treatment as [1 − (relative survival in the dsvATPase treatment/mean relative survival in the dsCON treatment)] × 100.

### 2.13. Statistical Analysis

All statistical analyses were performed using ANOVA with the PROC GLM procedure in SAS v9.4 [23]. Mortality data were arcsine-transformed prior to analysis to meet assumptions of normality and homogeneity of variance. All experiments were carried out with three independent biological replicates, and results are presented as mean ± standard error (SE), generated using GraphPad Prism version 8.0.1.

## 3. Results

### 3.1. SPc Formulation Enhances the Entry of the Recombinant Vector into Sf9 Cells

**Figure 1:**
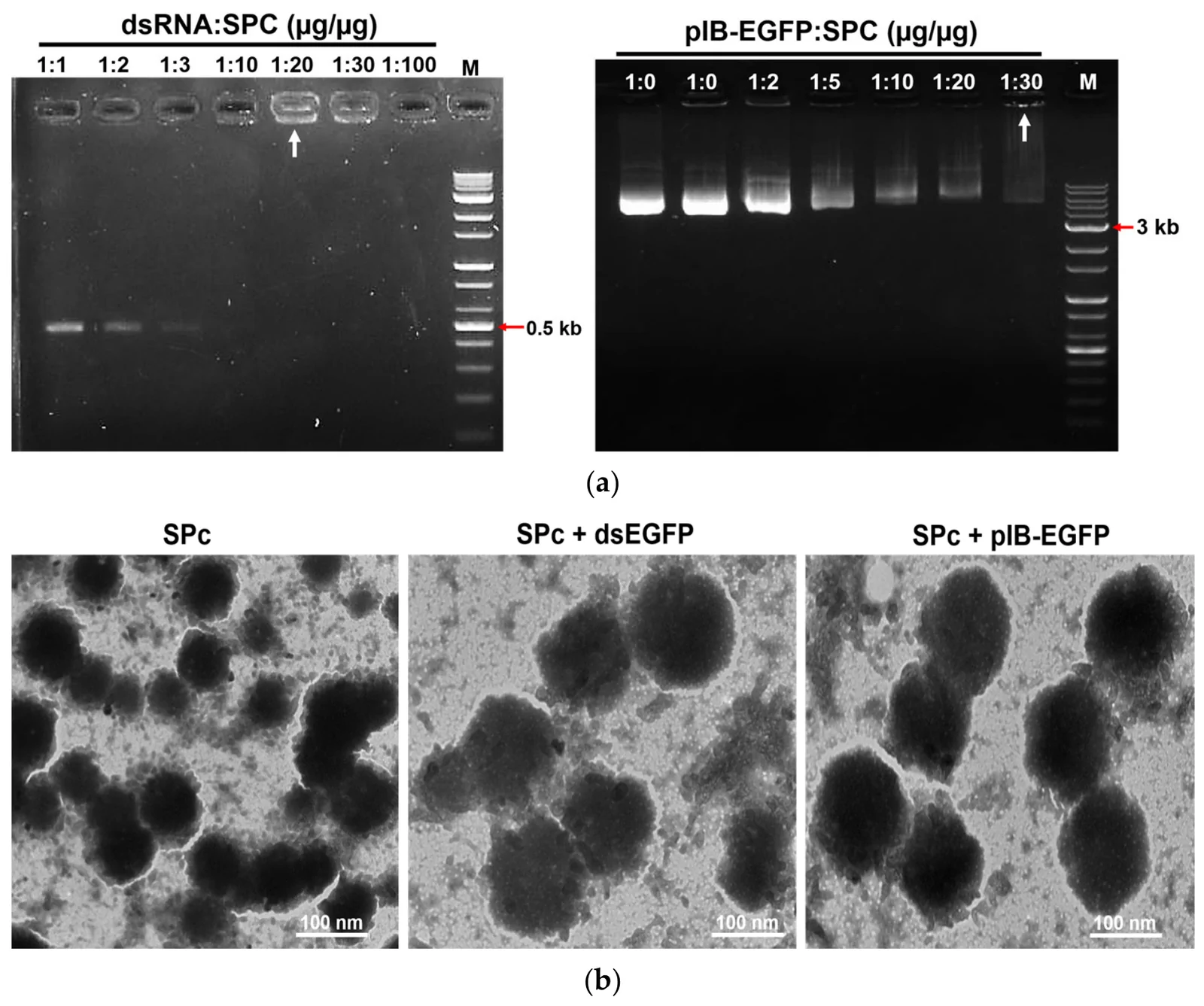

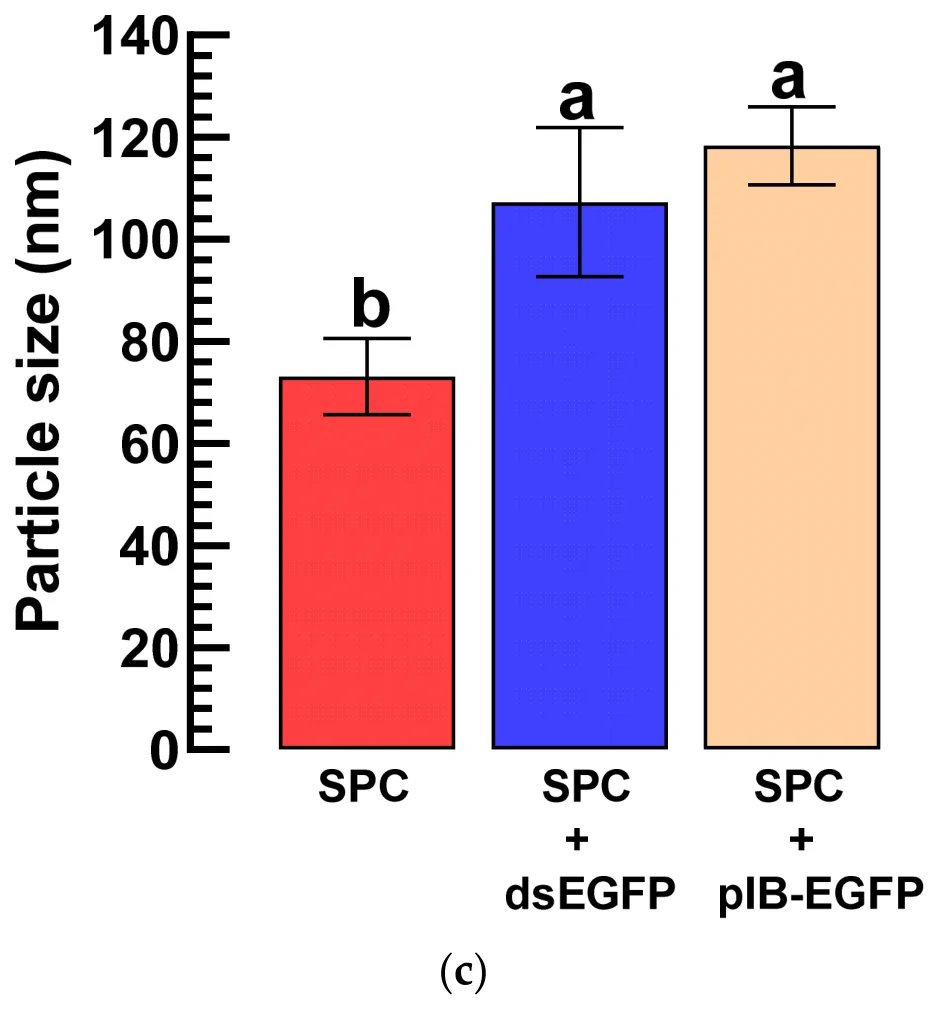
Characterization of SPc-mediated nucleic acid complex formation. (**a**) Agarose gel retardation assays showing the complexation of star polycation (‘SPC’) with dsRNA and pIB-EGFP plasmid DNA at the indicated mass ratios. Reduced electrophoretic migration and retention of nucleic acids in the loading wells indicate the formation of SPc–nucleic acid complexes. White arrows indicate the saturated complexes in the relative ratios. ‘M’ represents the 1 kb molecular marker. (**b**) TEM images showing the morphology of SPc particles and SPc-formulated dsRNA nanoparticles. Scale bars are indicated in each micrograph. (**c**) Particle-size analysis of SPc alone and SPc-formulated dsRNA/pIB nanoparticles. Bars represent mean ± SE. Different letters above bars indicate significant differences between treatments.

A recombinant vector (=pIB-EGFP) or dsRNA (443 bp) was formulated with SPc at a mass ratio of 1:20 (dsRNA:SPc) or 1:30 (pIB:SPc) (Figure 1a). With increases in the vector or dsRNA amounts, excessive DNA or dsRNA was visible on the gel at the expected sizes. The formulation was observed under TEM (Figure 1b). Compared with the unformulated 9~75 nm), the formulated (~100 nm) particle size increased almost 1.5-fold (Figure 1c).

**Figure 2:**
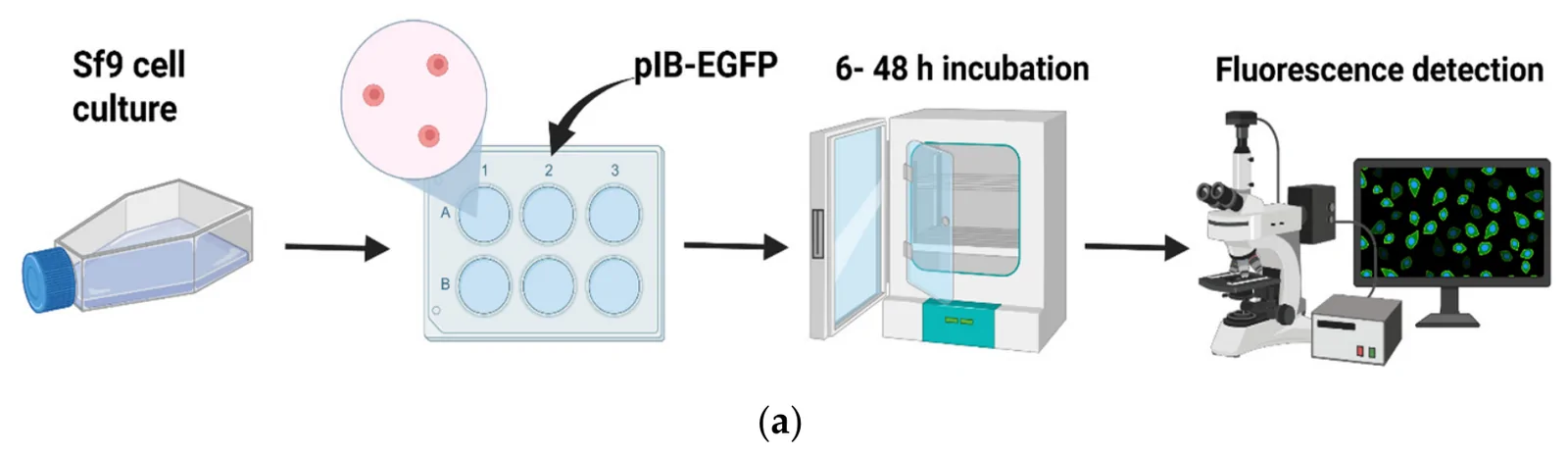

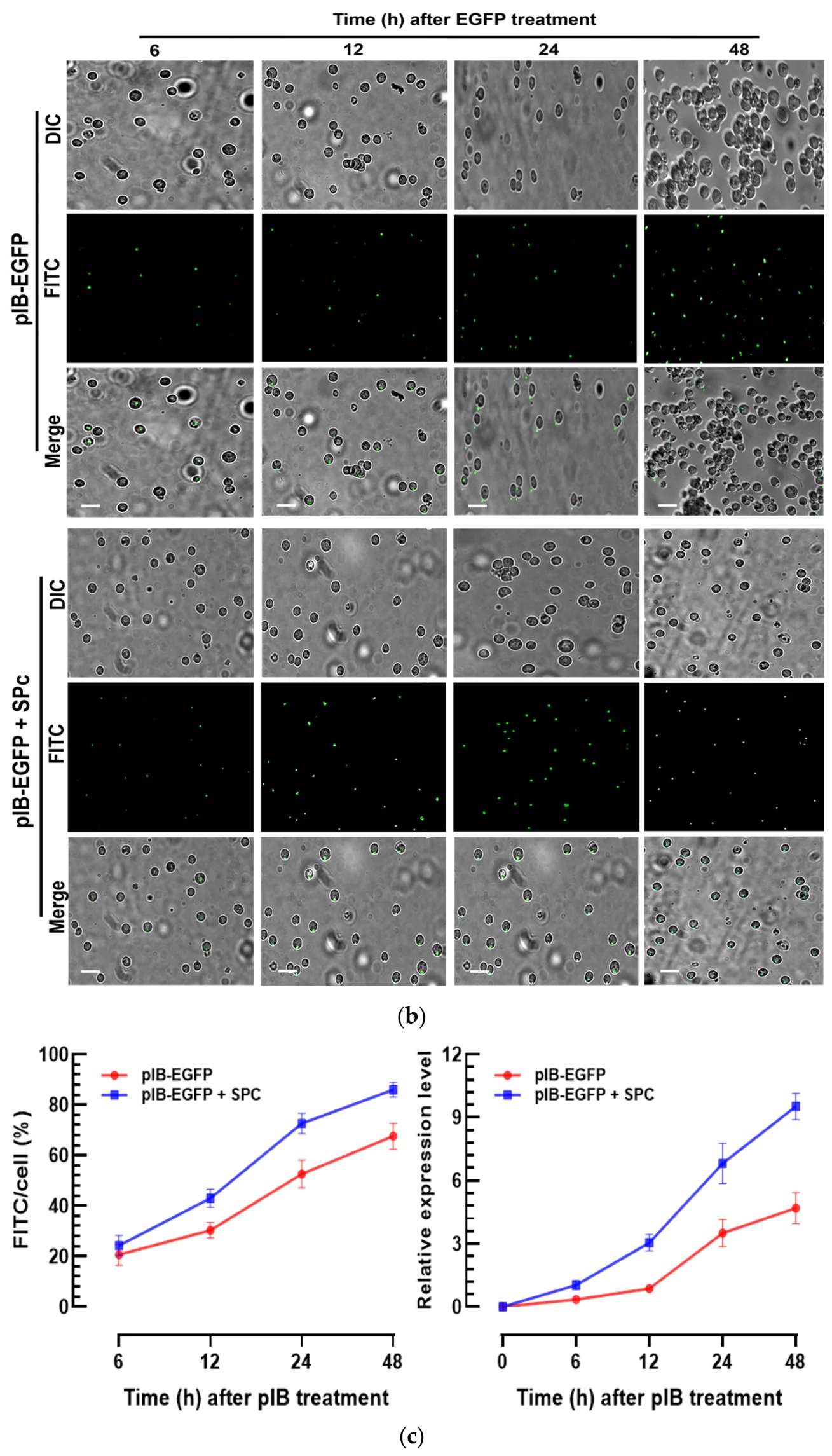
SPc formulation enhances pIB-EGFP delivery and transient *EGFP* expression in Sf9 cells. (**a**) Schematic representation of the in vitro delivery assay. Sf9 cells were treated with pIB-EGFP either alone or formulated with SPc, incubated for the indicated periods, and examined by fluorescence microscopy. (**b**) Representative differential interference contrast (‘DIC’), FITC fluorescence, and their combined (‘Merge’) images of Sf9 cells at 6–48 h after treatment with naked pIB-EGFP or SPc-formulated pIB-EGFP. (**c**) Quantification of FITC-positive cells and relative *EGFP* expression levels after pIB-EGFP treatment using qPCR. FITC-positive cells were quantified using ImageJ and expressed as a percentage of the total cell number. Data are presented as the mean ± SE from independent biological replicates. Each experiment was performed independently three times (*n* = 3), with one independently treated Sf9 cell-culture flask serving as one biological replicate.

The entry efficiency of the SPc formulation was assessed using a pIB vector expressing *EGFP* in Sf9 cells (Figure 2a). Compared with the unformulated vector, the formulated vector significantly increased the expression levels by exhibiting a high fluorescence intensity (Figure 2b). Quantification of the fluorescence showed that the formulation enhanced the entry of the vector into the cells regarding time and subsequent expression level (*F* = 28.5; df = 1, 28; *p* = 0.0059) (Figure 2c).

### 3.2. SPc Formulation Enhances IVTE and RNAi Efficiency in S. exigua

**Figure 3:**
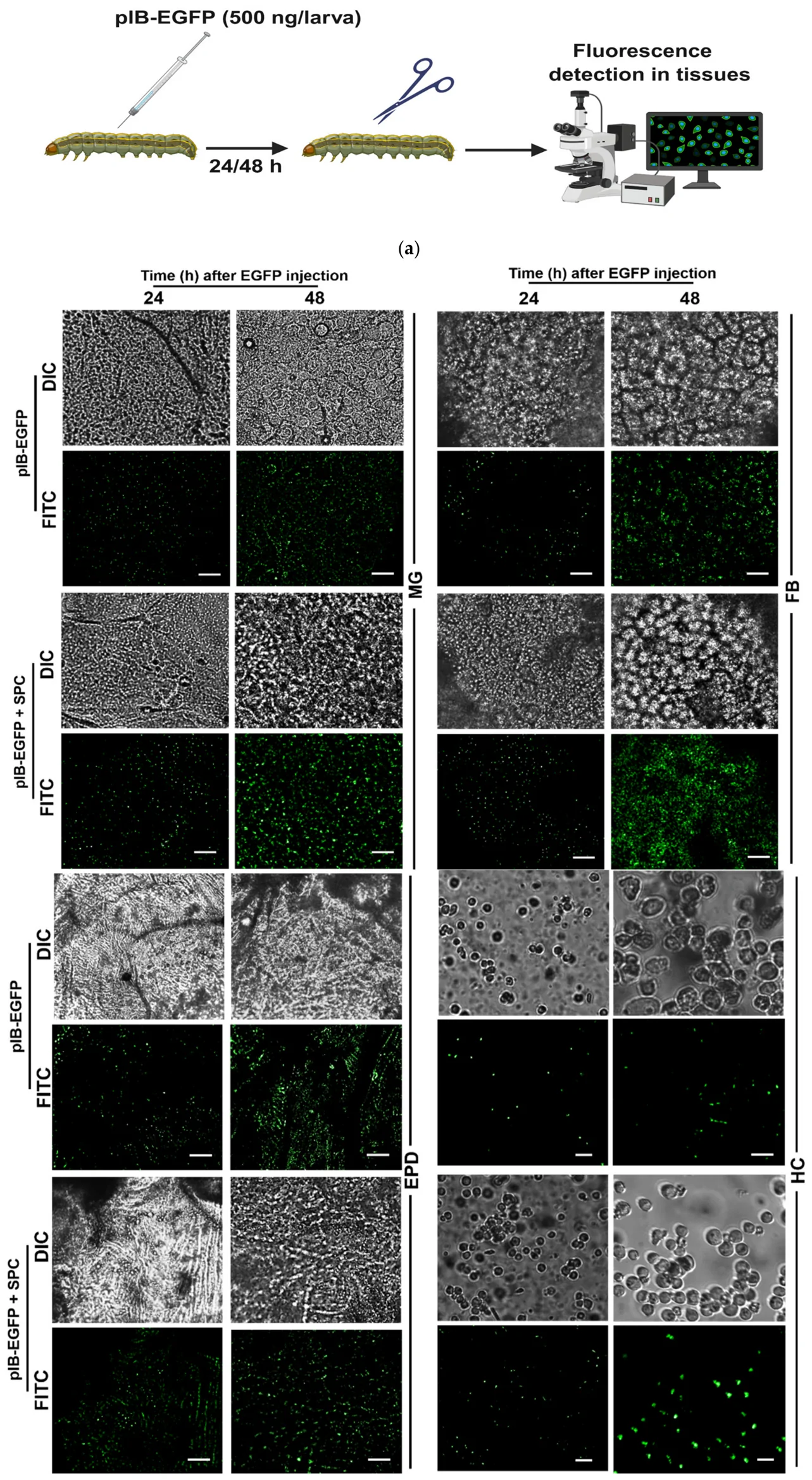

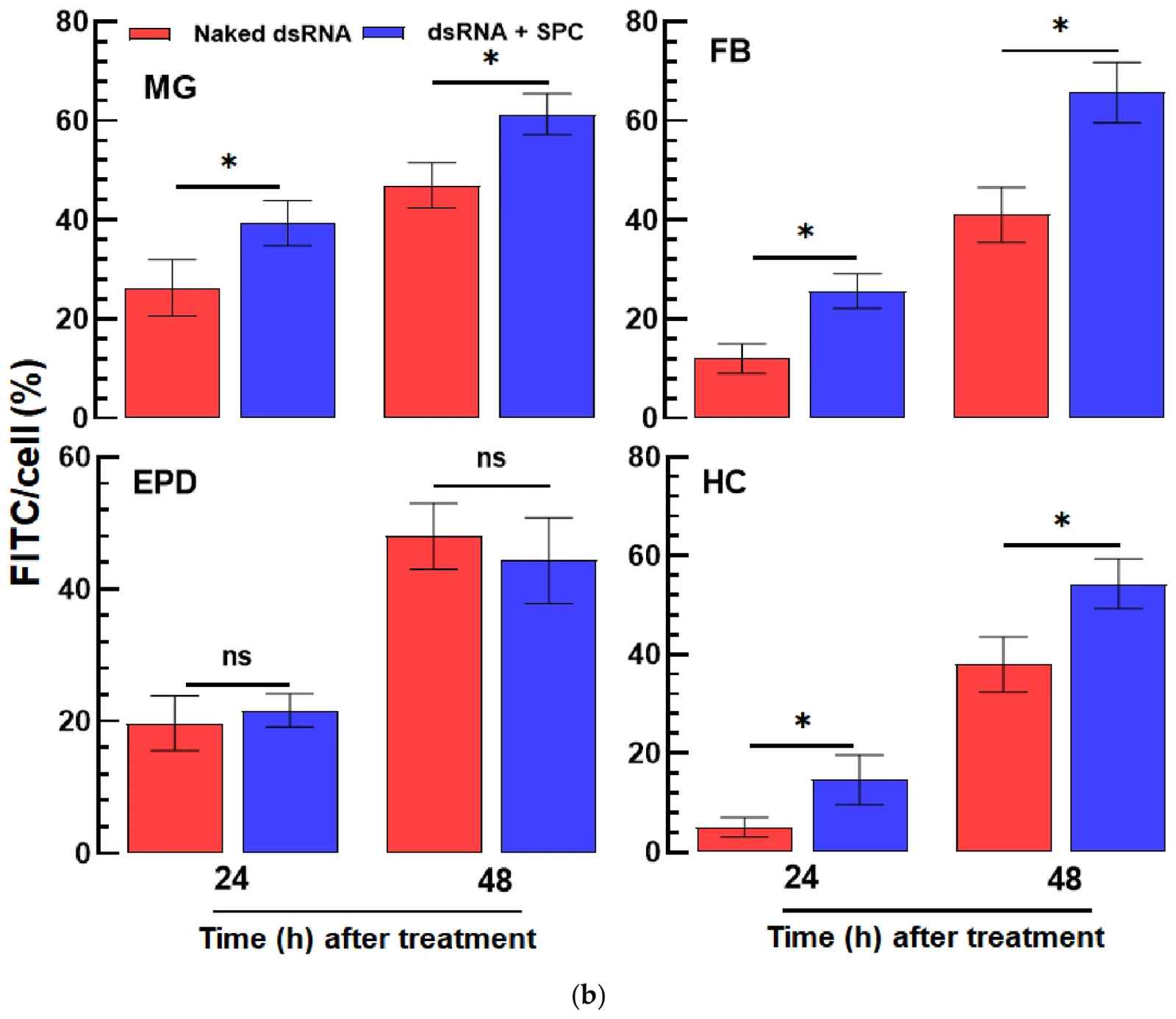
SPc formulation improves IVTE of *EGFP* reporter gene in multiple tissues of *S. exigua*. (**a**) Schematic overview of the IVTE assay. L5 larvae of *S. exigua* were injected with pIB-EGFP or SPc-formulated pIB-EGFP, and tissues were dissected at 24 and 48 h after injection for fluorescence detection. (**b**) Representative DIC and FITC fluorescence images showing EGFP expression in the midgut (‘MG’), fat body (‘FB’), epidermis (‘EPD’), and hemocytes (‘HC’). Scale bars represent 10 μm. Fluorescence intensity was quantified using ImageJ and expressed as the mean FITC fluorescence per cell, calculated by dividing the total FITC signal by the total number of cells. Each treatment was independently replicated three times (*n* = 3), with one independently treated L5 larva serving as one biological replicate. Asterisks above standard error bars indicate significant differences between means at Type I error = 0.05 (LSD test). ‘ns’ represents no significant difference.

The enhanced entry of the recombinant vector by the SPc formulation suggested its application to IVTE for the target expression in insects. When the formulated vector was injected into the hemocoel of *S. exigua* larvae, the expression detected by fluorescence was observed in different tissues, namely, the midgut, fat body, epidermis, and hemocytes (Figure 3a).

With an increase in the incubation time after the injection, the expression levels increased in all four test tissues. The SPc formulation significantly increased the IVTE levels in most tissues, except the epidermis (Figure 3b).

**Figure 4:**
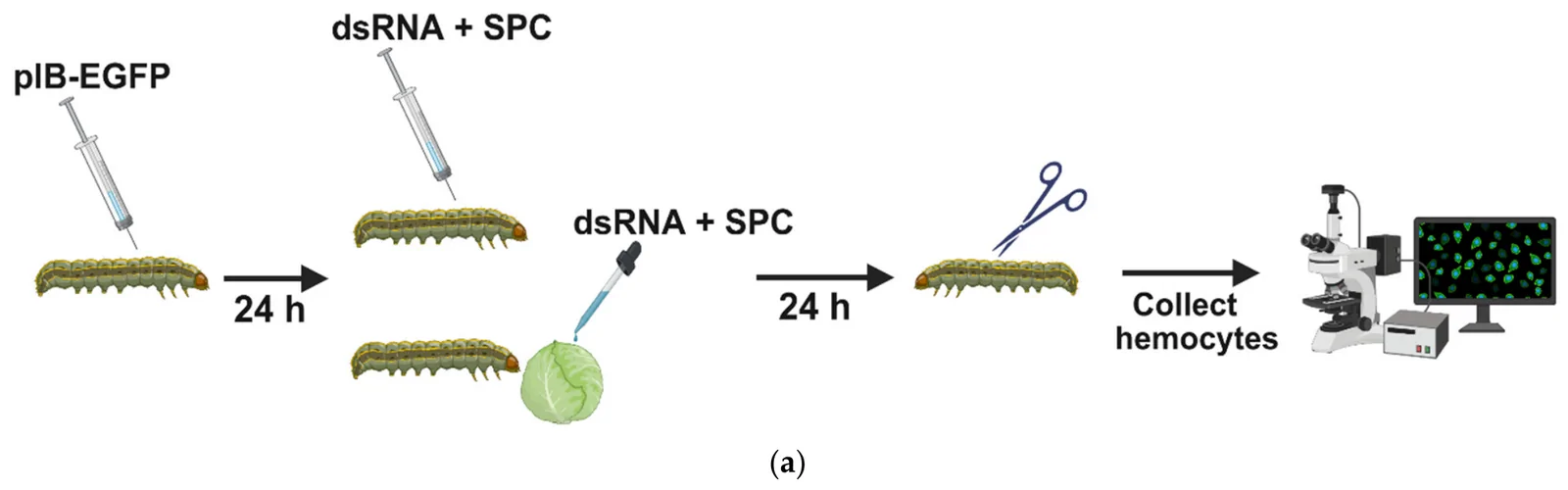

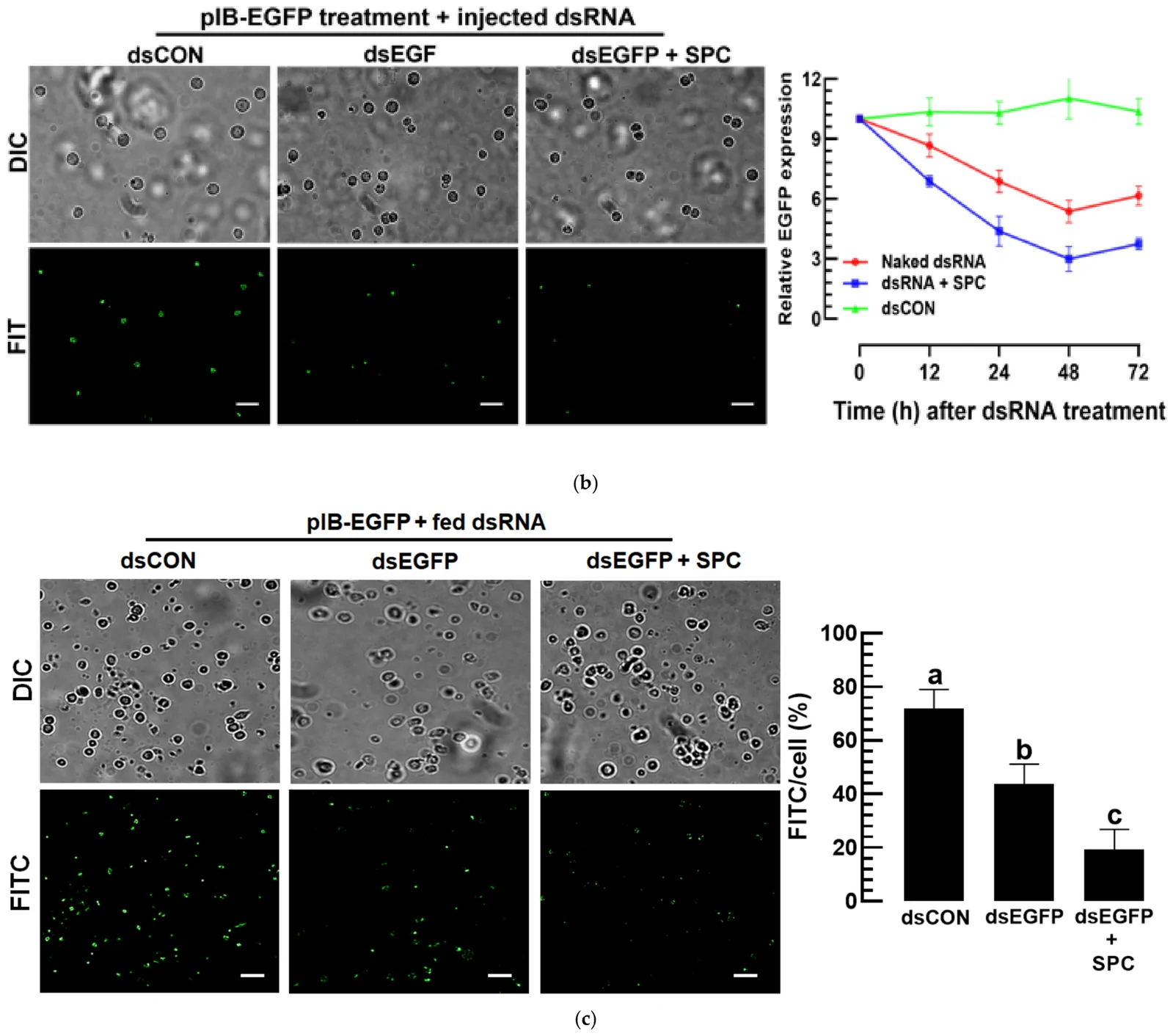
SPc formulation enhances dsRNA-mediated RNA interference against *EGFP* IVTE expression in *S. exigua*. (**a**) Experimental design for evaluating the RNAi efficiency after transient *EGFP* expression. A total of 500 ng of pIB-EGFP were injected into L5 larvae. Larvae expressing *EGFP* were then treated with dsEGFP either by injection (200 ng/µL) or oral feeding (500 µg/mL), with or without SPc formulation, and hemocytes were collected for fluorescence and expression analyses. (**b**) Effects of injected dsEGFP on EGFP fluorescence and transcript abundance. Fluorescent signals were shown by FITC mode using a fluorescence microscope. Scale bars represent 10 μm. The line graph shows the relative EGFP expression over time after dsRNA treatment. Each treatment was independently replicated three times (*n* = 3), with one independently treated L5 larva serving as one biological replicate. (**c**) Effects of orally delivered dsEGFP (500 µg/mL) on fluorescence. FITC-positive cells were quantified using ImageJ and expressed as a percentage of the total cell number. Scale bars represent 10 μm. The control of the dsRNA treatment (dsCON) represents no dsRNA treatment. Each treatment was independently replicated three times (*n* = 3), with one independently treated L5 larva serving as one biological replicate. Different letters above standard error bars indicate significant differences between means at Type I error = 0.05 (LSD test).

Under the IVTE of EGFP, its specific dsRNA was injected or fed to the larvae to test the effect of the SPc formulation on enhancing the RNAi efficiency (Figure 4a). Compared with the unformulated vector (dsEGFP), the formulated dsRNA (dsEGFP + SPC) significantly (*F* = 18.8; df = 1, 18; *p* = 0.0001) increased the RNAi efficiency when the dsRNAs were injected (Figure 4b). The RNAi efficacies were also observed when the dsRNAs were fed to larvae, in which the SPc formulation significantly enhanced the RNAi efficiency compared with the unformulated vector (Figure 4c).

### 3.3. SPc Formulation Enhances the Insecticidal Activity of dsRNA Against Two Lepidopteran Insects

**Figure 5:**
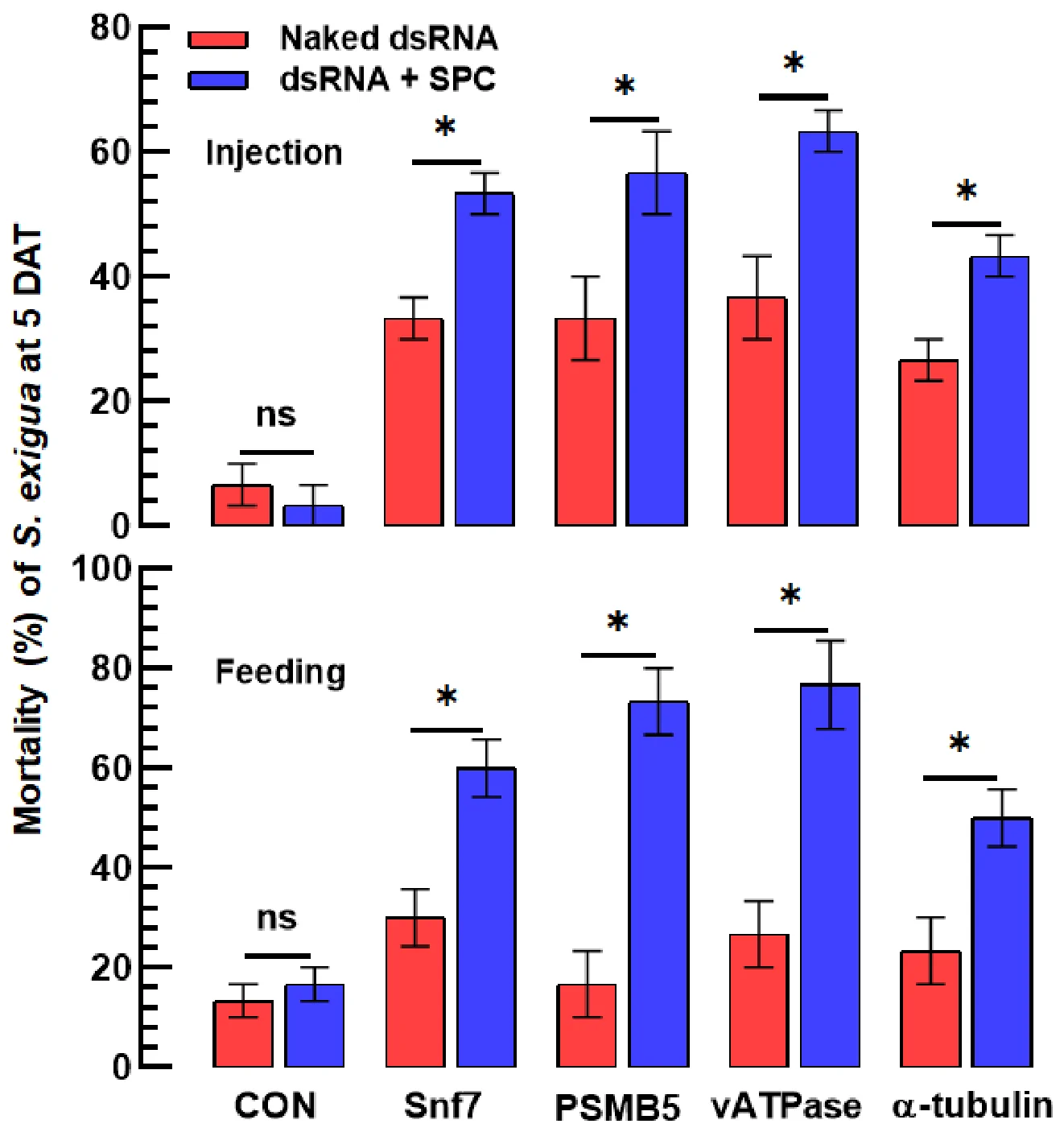

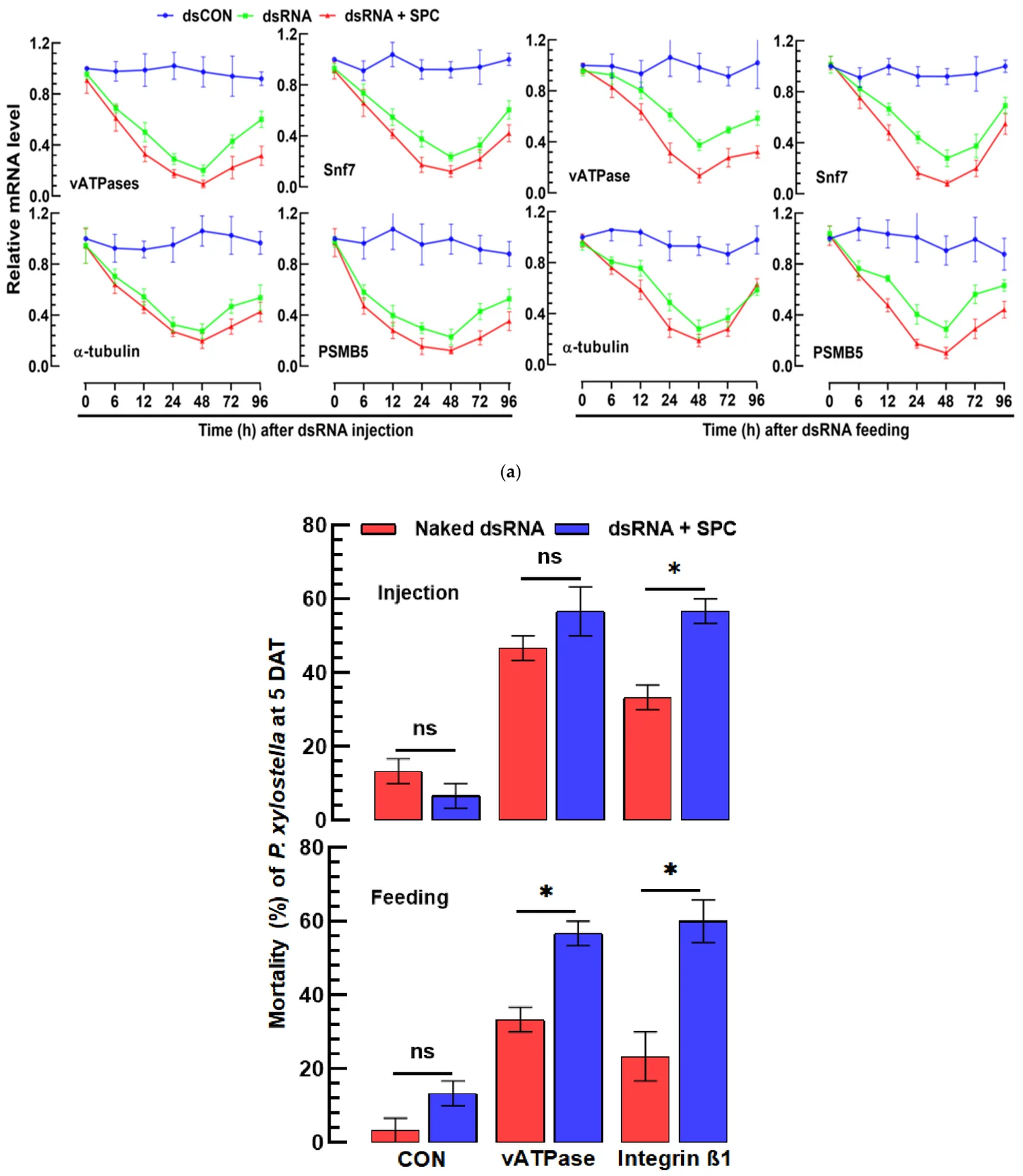

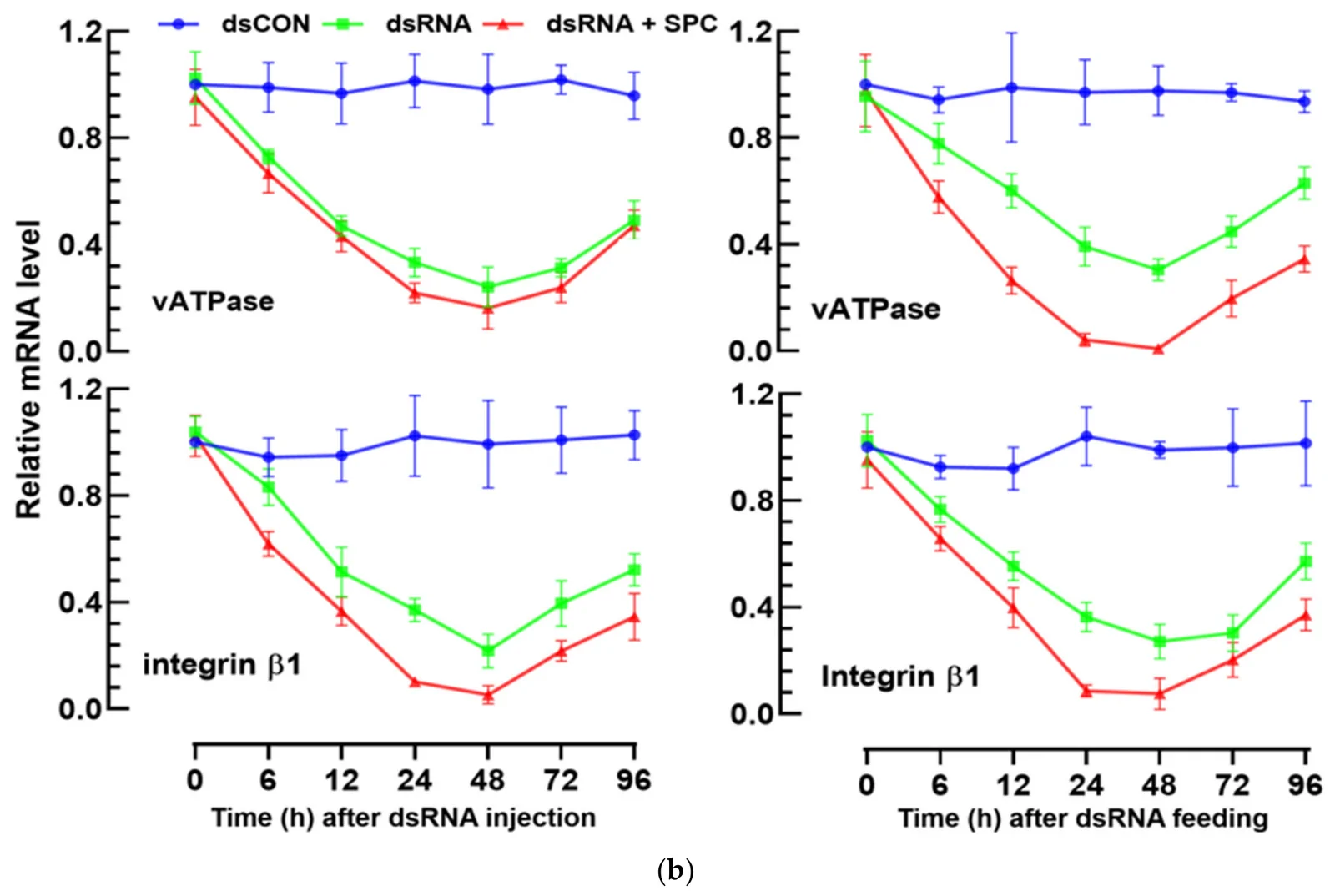
SPc formulation increases insecticidal activity of dsRNA in lepidopteran insects. (**a**) RNAi-mediated mortality and target-gene knockdown in *S. exigua*. Larvae were treated by injection (200 ng/larvae) or feeding (500 µg/mL) with naked dsRNA or SPc-formulated dsRNA at a dsRNA:SPc mass ratio of 1:20, targeting *Snf7*, *PSMB5*, *vATPase*, or *α-tubulin*. Mortality was recorded at 5 days after treatment (‘DAT’), and transcript levels were quantified by RT-qPCR over time using specific primers (Table S1). dsRNA specific to *GFP* (‘dsCON’) served as the control RNAi. Each treatment was independently replicated three times (*n* = 3), with 10 larvae per biological replicate. (**b**) RNAi-mediated mortality and target-gene knockdown in *P. xylostella*. Larvae were treated with dsRNAs targeting *vATPase* or integrin β1 by injection or feeding. Transcript levels were quantified by RT-qPCR over time using specific primers (Table S1). Asterisks indicate significant differences between naked dsRNA and SPc-formulated dsRNA; ‘ns’ indicates no significant difference. Each bioassay was replicated three times with 10 larvae per replicate.

The enhanced RNAi efficiency due to the SPc formulation was applied to control insect pests. Two lepidopteran insects (*S. exigua* and *P. xylostella*) were used to test this effect. Test dsRNAs specific to four target genes selected from previous studies were formulated with SPc and applied to the larvae of S. exigua by injection or feeding (Figure 5a). All four dsRNAs exhibited significant RNAi efficacy without the formulation. However, the SPc formulation significantly increased the RNAi efficacy and subsequent mortalities compared with the unformulated vector. Even though feeding dsRNAs showed low control efficacies compared with dsRNA injections, the SPc formulation significantly enhanced the control efficacy of the feeding application to a level that was almost comparable with that of the injection application. In *P. xylostella*, two genes were targeted with their specific dsRNAs (Figure 5b). Both dsRNAs exhibited RNAi efficacies in either feeding or injection applications, in which the SPc formulation increased the RNAi efficiencies and subsequent insecticidal activities. Overall, SPc formulation enhanced the RNAi efficiency of dsRNA with feeding application and increased the insecticidal activity against lepidopteran insect pests.

### 3.4. SPc Formulation Enhances the Insecticidal Activity of dsRNA Against Aphid and Thrips

**Figure 6:**
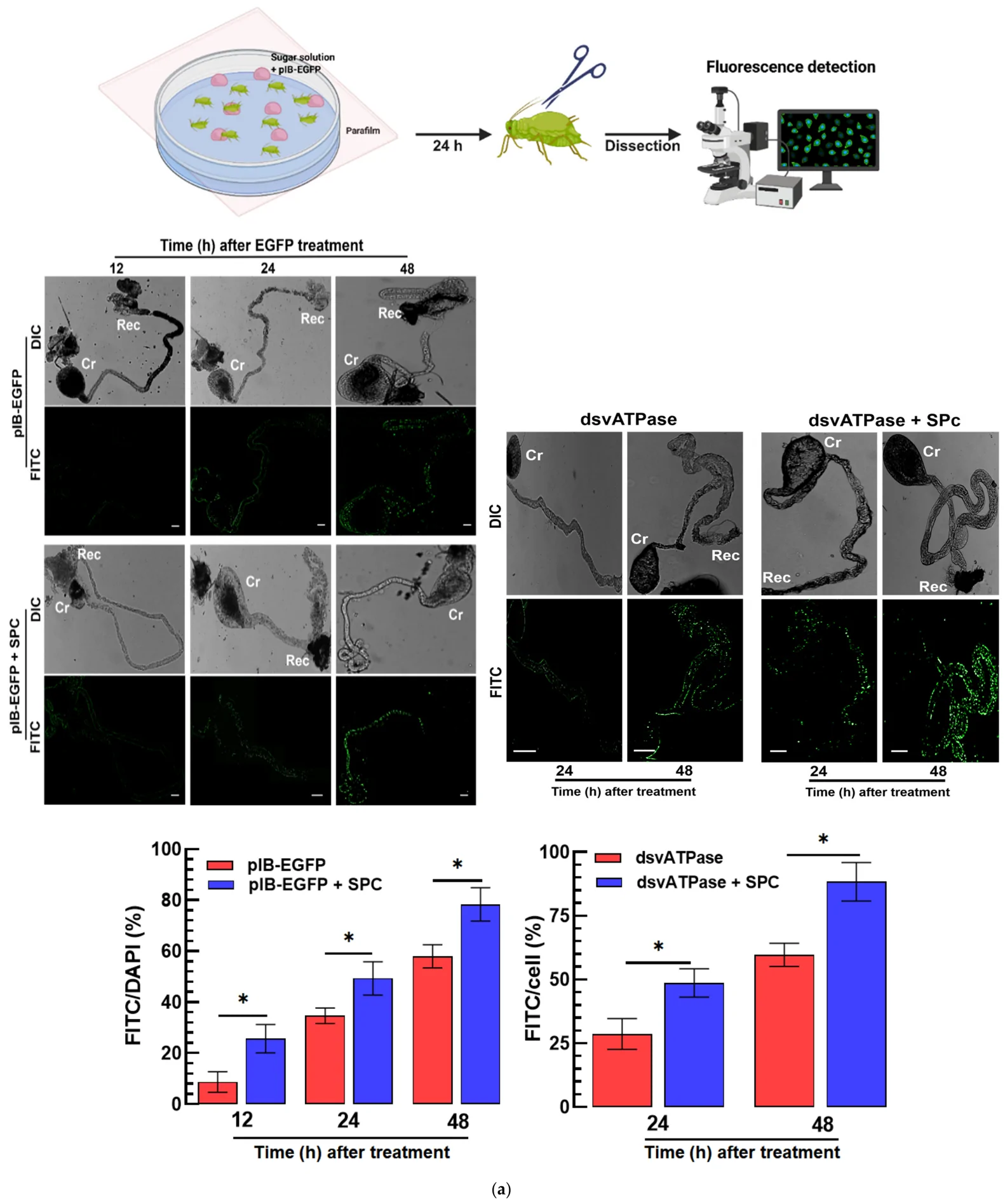

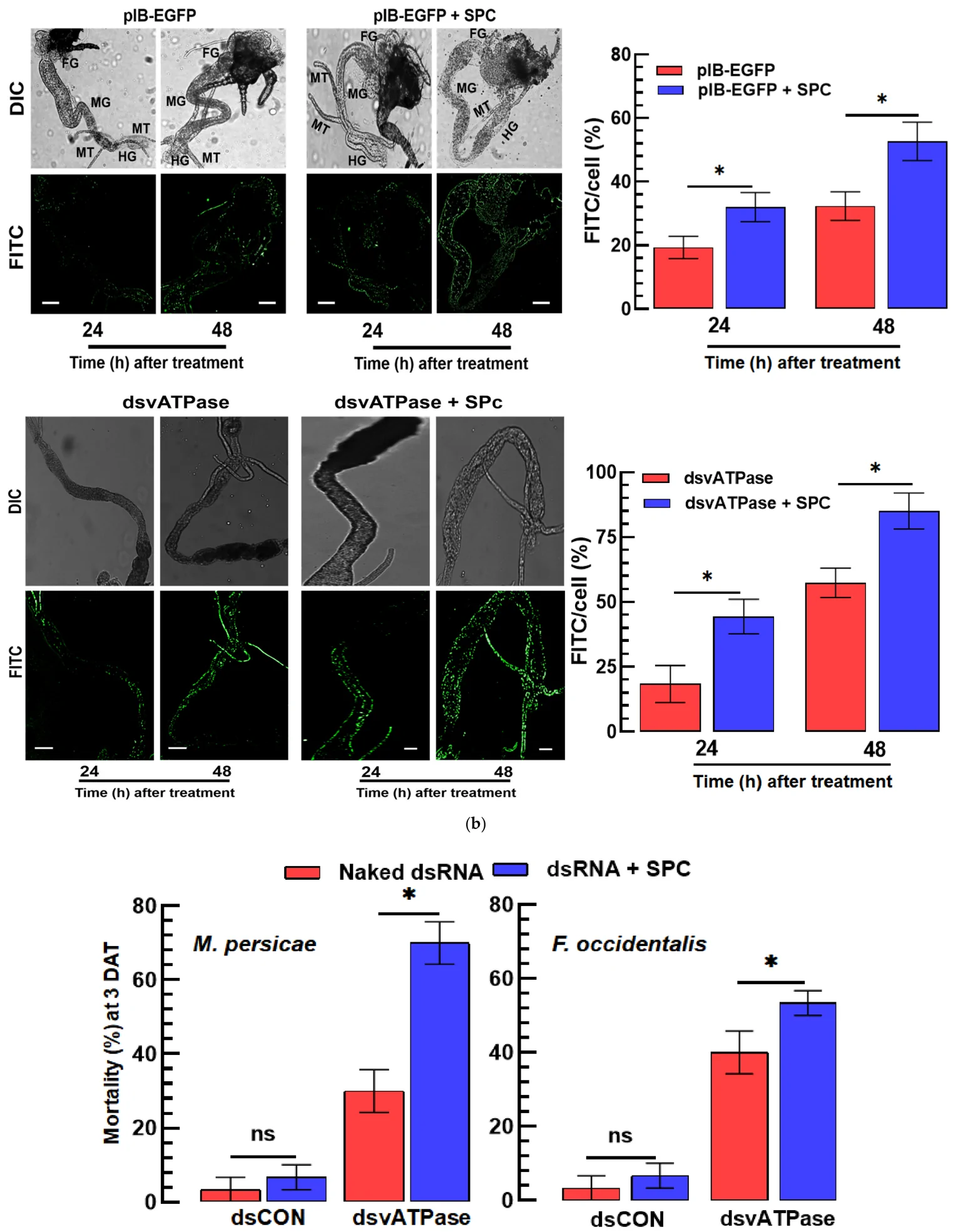

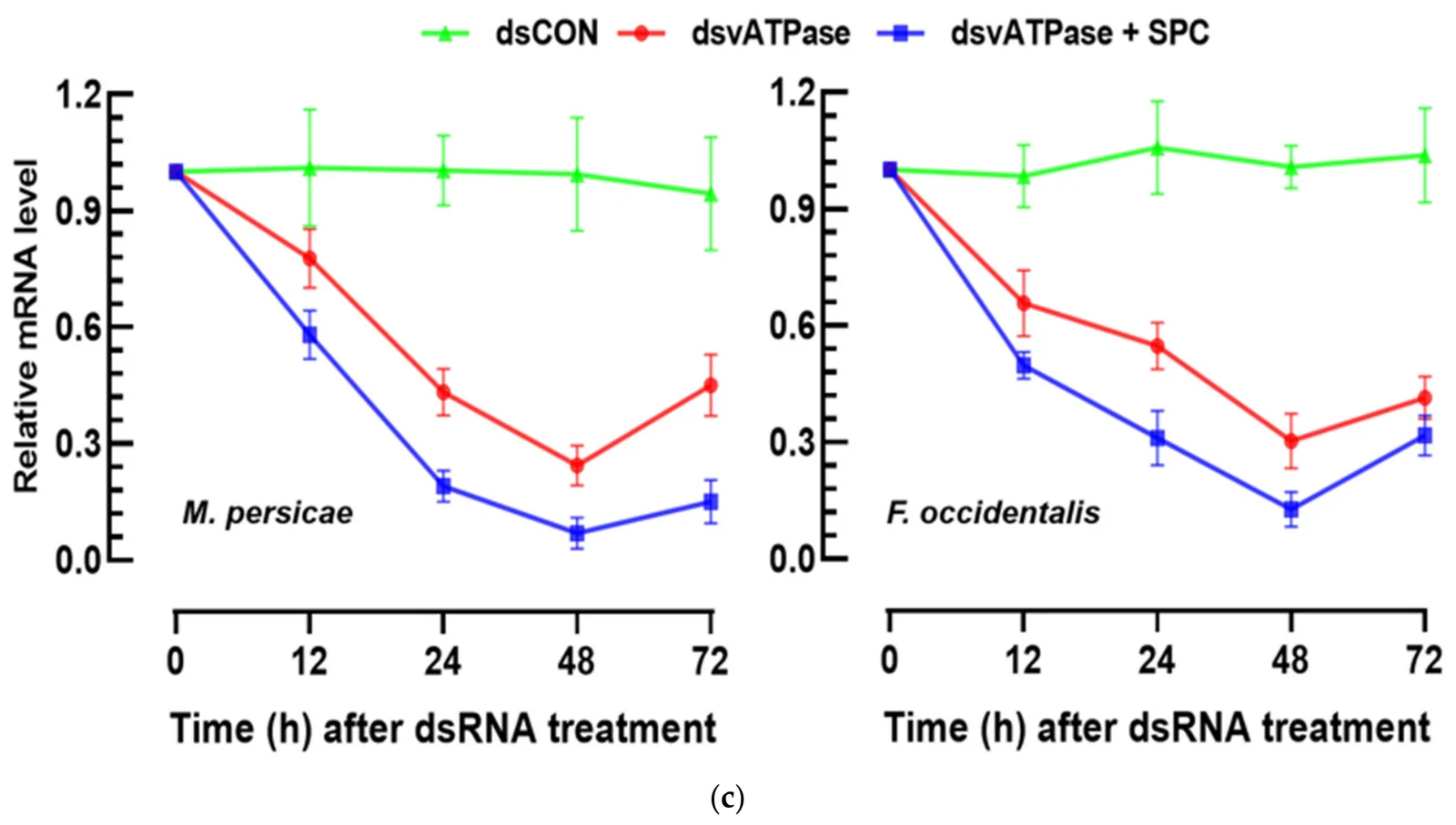
SPc formulation enhances oral nucleic acid delivery and RNAi efficacy in sucking insect pests. (**a**) Oral delivery of pIB-EGFP/dsRNA to *M. persicae* using a parafilm-based feeding assay. Dissected aphid tissues were observed in differential interference contrast (‘DIC’) mode. Fluorescent signals were observed in FITC mode using a fluorescence microscope at 12, 24, and 48 h after treatment with naked pIB-EGFP or SPc-formulated pIB-EGFP. Fluorescence intensities were analyzed using ImageJ software. FITC intensity normalized by the DAPI signal intensity. ‘Cr’ for crop and ‘Rec’ for rectum. Each treatment was independently replicated three times (*n* = 3), with one independently treated aphid serving as one biological replicate. (**b**) Oral delivery of pIB-EGFP to *F. occidentalis*. Dissected tissues, namely, foregut (‘FG’), midgut (‘MG’), hindgut (‘HG’), and Malpighian tubules (‘MT’), were observed in DIC mode. Fluorescent signals were observed in FITC mode using a fluorescence microscope at 24 and 48 h after treatment with naked pIB-EGFP or SPc-formulated pIB-EGFP. FITC intensity normalized by the DAPI signal intensity. Each treatment was independently replicated three times (*n* = 3), with one independently treated larva serving as one biological replicate. Scale bars represent 100 μm. (**c**) RNAi-mediated mortality at 3 days after treatment (‘DAT’) and *vATPase* knockdown in *M. persicae* and *F. occidentalis* following oral delivery of naked (500 µg/mL) or SPc-formulated dsvATPase (1:20 mass ratio). dsRNA specific to *GFP* (‘dsCON’) served as the control RNAi. Transcript levels were quantified by RT-qPCR over time using specific primers (Table S1). Each bioassay was replicated three times with 10 insects per replicate. Asterisks above the standard error (SE) bars indicate significant differences between two means at Type I error = 0.05 (Student’s *t*-test). ‘ns’ represents no significant difference.

Enhanced delivery of nucleic acids by SPc formulation was tested in two sucking insects: aphid (*M. persicae*) and thrips (*F. occidentalis*). Feeding the pIB construct expressing *EGFP* to the aphids caused its expression in the gut epithelium, in which the SPc formulation further increased the expression levels, probably enhancing the vector delivery to the cells (Figure 6a). Similarly, the thrips were also fed the vector construct and expressed the *EGFP* gene in the gut epithelium, in which the SPc formulation increased the expression level (Figure 6b).

The enhanced delivery of the vector in the sucking insects suggested its application to dsRNA treatment to increase the control efficacy. dsRNA specific to *vATPase* was able to kill the aphids and thrips using oral administration (Figure 6c). The control efficacy was further enhanced by SPc formulation.

**Figure 7:**
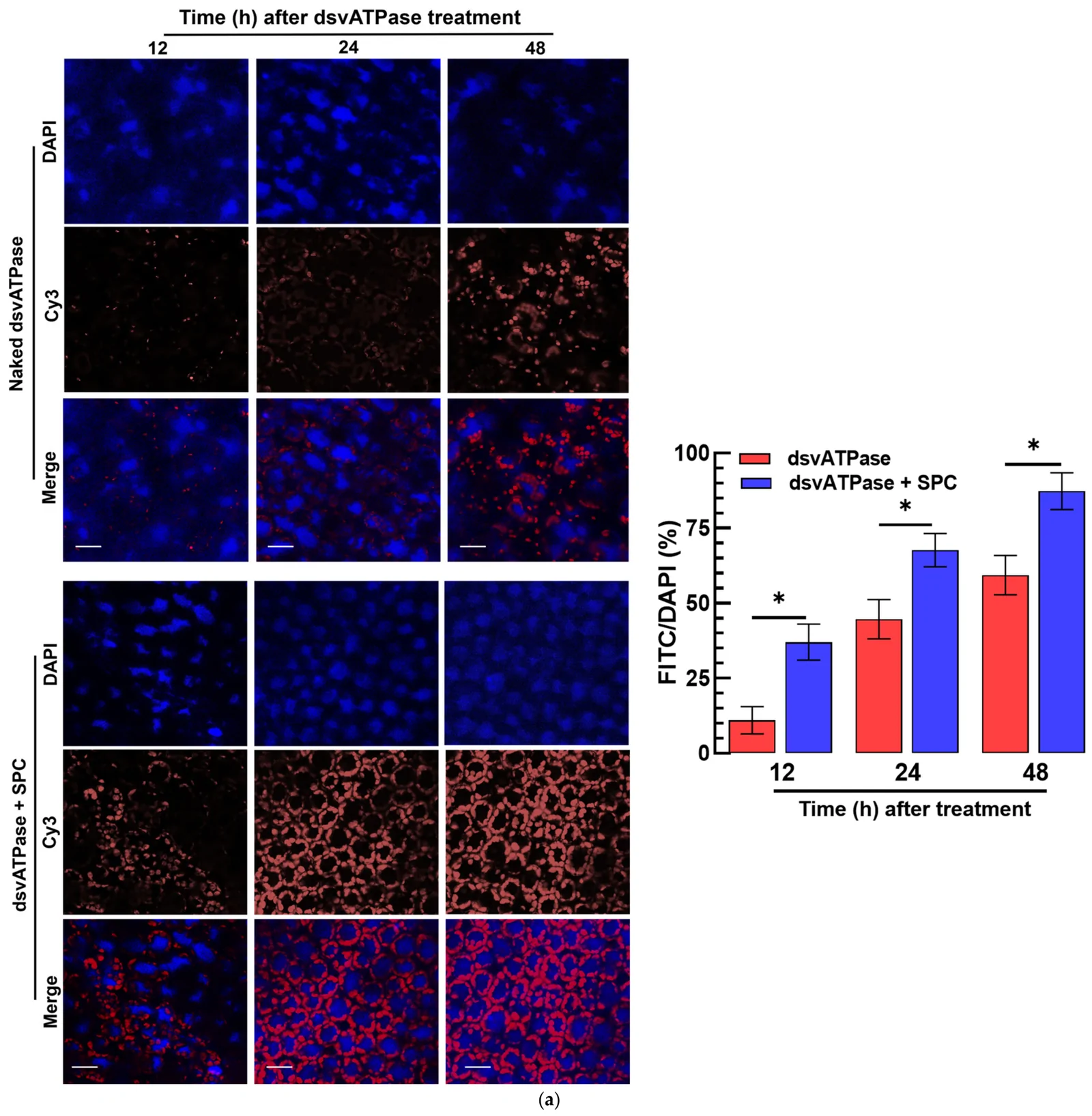

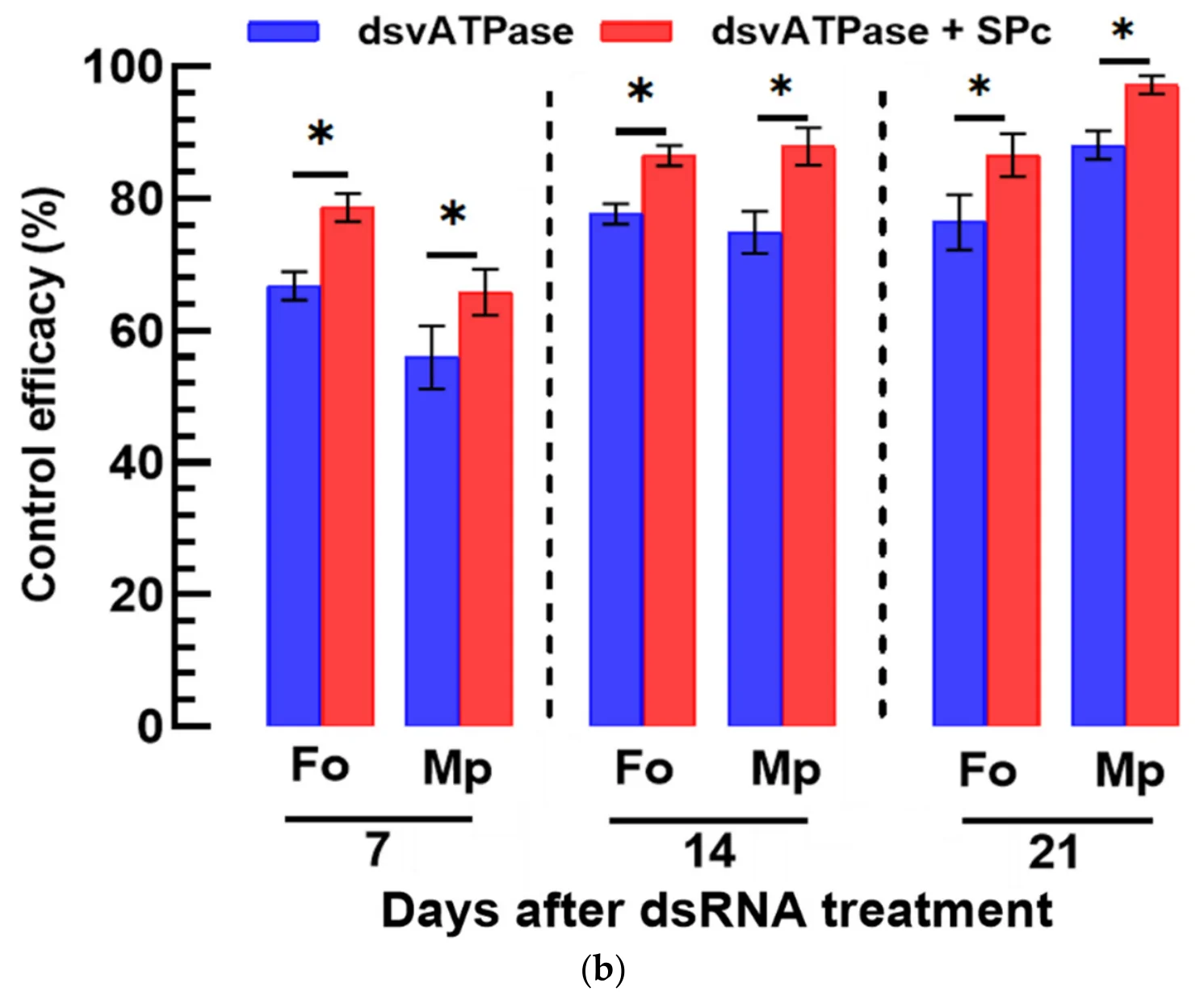
Foliar application of SPc-formulated dsRNA enhances dsRNA delivery into hot pepper leaves and improves control efficacy against aphids and thrips. (**a**) Translocation of Cy3-labeled dsRNA into hot pepper leaf tissues after foliar application. Leaves were treated with naked Cy3-dsRNA or SPc-formulated Cy3-dsRNA and examined at the indicated time points by fluorescence microscopy. SPc formulation enhanced dsRNA penetration and accumulation in leaf tissues compared with naked dsRNA. Each treatment was independently replicated using three plants (*n* = 3), with one plant serving as one biological replicate. Scale bars represent 10 µm. (**b**) Insecticidal activity of sprayed dsvATPase against *M. persicae* and *F. occidentalis*. Hot pepper plants were sprayed with naked or SPc-formulated dsvATPase, and insect mortality was assessed after feeding on treated plants. Control efficacy was evaluated using three independent hot pepper plants per treatment (*n* = 3), with each plant infested with 40–60 adult aphids or L1/L2 thrips larvae serving as one biological replicate. Bars represent mean ± SE. Asterisks indicate significant differences between naked and SPc-formulated dsRNA treatments.

To practically apply the SPc-formulated dsRNA to control the sucking insects, it was sprayed on hot peppers. The Cy3-labeled dsRNA entered the leaf tissues with an increase in the incubation time (Figure 7a). When the Cy3-dsRNA was formulated, its delivery was greatly enhanced compared with the unformulated vector. The entered dsRNAs were localized in the cytoplasm, as they were visualized in the merged photos. The SPc-formulated dsRNA specific to *vATPase* was sprayed on the hot peppers (Figure 7b). Both aphids and thrips significantly suffered from high mortalities, where the SPc formulation caused higher mortalities than the unformulated vector.

## 4. Discussion

dsRNA delivery to target cells is essential for efficient RNAi but hindered by degrading enzymes in the extracellular compartment, membrane hydrophobicity, and intracellular endosome formation. However, it can be improved by using a formulation. This study evaluated the efficacy of dsRNA delivery using an SPc nano-formulation. The SPc formulation increased the IVTE efficiency of a eukaryotic expression vector in Sf9 cells and insect tissues. It also enhanced the RNAi efficiency of the dsRNA construct in the cells and tissues. The increased RNAi efficiency resulted in high mortality against lepidopteran and sucking insects.

The enhanced delivery of nucleic acids by SPc formulation was validated by IVTE using Sf9 cells. The entry of the DNA construct was much improved by the SPc formation. The GFP gene expression encoded in the expression vector suggests that the DNA construct entered the nucleus, which was facilitated by the formulation. SPc is a highly efficient gene vector into live cells through a transdermal delivery system [18]. It was applied for efficient gene silencing and effective control toward aphids [24]. Subsequent application of SPc is limited to drug/dsRNA delivery for controlling plant diseases and pests [25]. Later, SPc was applied to construct a microRNA delivery platform for plants [21]. There are at least two pathways for dsRNA to enter cells through a channel—systemic RNAi deficiency (SID) or endocytosis—where the latter is further classified into clathrin-dependent endocytosis, caveolin-dependent endocytosis, and macropinocytosis [26,27]. In insects, clathrin-dependent endocytosis is a main route for dsRNA cell entry, but other pathways operate depending on the tissues involved [15]. Indeed, Ma et al. [28] demonstrated that SPc enhances dsRNA delivery in *S. frugiperda* by activating clathrin-mediated endocytosis. In addition to the RNA construct delivery, our current result suggests the SPc formulation facilitates the DNA construct entrance into the nucleus.

The RNAi efficiency was much improved by the SPc formulation in larvae of *S. exigua*, as well as the Sf9 cell line. These lepidopteran insects have been regarded as poor targets for RNAi, where the RNAi efficiency fluctuates in different species, stages, and other environmental conditions [29]. Later, the reluctance of Sf9 cells against dsRNA is explained by the endosome formation leading to a lysosomal degradation pathway [30]. In contrast, the beetle *Tribolium cataneum* is highly susceptible to dsRNA and usually exhibits high RNAi efficiencies [31]. One factor for the high RNAi efficiency in coleopteran insects is explained by endosome escape of dsRNA because they possess a unique dsRNA-binding protein called Staufen C, which helps dsRNA exit into the cytoplasm via the ERAD (Endoplasmic Reticulum-Associated Degradation) pathway [16]. SPc protects dsRNA from degradation by RNase, which increases the stability of dsRNA in the hemolymph and promotes endosome escape of the dsRNA in the cells [28]. Although the lepidopteran insects do not have Staufen C, the enhanced RNAi efficiency in the lepidopteran insects can be explained by the stability and endosome escape, along with the SPc formulation in our current study.

The SPc formulation enhanced the RNAi against two sucking insects: thrips and aphids. The enhanced RNAi can be explained by the enhanced entry of dsRNA into the sucking insects and their host plant. However, these two sucking insects are slightly different in feeding behavior. Aphids feed through phloem of the host plants while thrips exhibit sucking behavior of cell contents exuding after breaking the plant mesophyll with its mandible [32]. Thus, the SPc formulation effect on RNAi efficiency compared with naked dsRNA treatment was greater in aphids than in thrips. It is noticeable that the sprayed dsRNA without any formulation on hot peppers caused relatively significant mortality of aphids in laboratory conditions. This suggests the sprayed dsRNA presumably enters the plant cells via stomata rather than through the cuticle layer. Even though this event may not occur due to desiccation or UV breakdown in field conditions, it suggests that SPc formulation may facilitate the dsRNA entry to plant tissues through the stomata by protecting dsRNA and releasing it gradually. This hypothesis needs to be explored in future study.

Altogether, our study evaluated the effect of SPc nanoparticles in enhancing DNA/RNA delivery to insects and their host plant. It was found that their high efficiency in dsRNA delivery to target cells led to high insect mortality, supporting the application of SPc formulation for practical pest control against lepidopteran and sucking insects.

## Data Availability

The original contributions presented in the study are included in the article/Supplementary Materials; further inquiries can be directed to the corresponding author.

## Supplementary Materials

The following supporting information can be downloaded at: https://www.mdpi.com/article/10.3390/insects17080869/s1, Table S1. Sequences of primers and probes used in this study.
