# Supplementary material for: Enhanced Delivery of Nucleic Acids to Insect Cells by Star Polycation Formulation
Source: Insects. 2026 Aug 20;17(8):869. doi: 10.3390/insects17080869 (PMC13513597; doi:10.3390/insects17080869)
Supplement: Supplementary file 1 [file insects-17-00869-s001.zip › insects-4486758-supplementary.pdf]

Table S1. Sequences of primers used in this study

Primer	application	Orientation	Sequence (5'-3')	Amplicon (bp)
**T7-dsEGFP**	dsRNA	Forward	TAATACGACTCACTATAGGGGTAAACGGCCACAAGTTCAG	443
		Reverse	TAATACGACTCACTATAGGGTGTCGGCCATGATATAGACG	
**EGFP-ORF**	Full ORF amplification	Forward	ATTGTCGACATGGTGAGCAAGGGCG	738
		Reverse	GCCCTGCAGTTACTTGTACAGCTCGTCC	
**Fo-vATPase B**	qPCR	Forward	GATGAATTCTATTGCTCGTG	317
		Reverse	ACTCAGCAGCAGTGAGAGCC	
**Fo-EF1α**	qPCR	Forward	TCAAGGAACTGCGTCGTGGAT	160
		Reverse	ACAGGGGTGTAGCCGTTAGAG	
**Se-Snf7**	qPCR	Forward	GCCAAAGGGATGAGATCCACA	166
		Reverse	CACCGACTTGGTACGACTCC	
**T7-Se-Snf7**	dsRNA	Forward	TAATACGACTCACTATAGGGAGAGCCAAAGGGATGAGATCCACA	212
		Reverse	TAATACGACTCACTATAGGGAGACACCGACTTGGTACGACTCC	
**Se-PSMB5**	qPCR	Forward	GGCAAAGTGTTTAGCGTGGG	149
		Reverse	ATCGCTGCAATCTTCGTTGC	
**T7-Se-PSMB5**	dsRNA	Forward	GGCAAAGTGTTTAGCGTGGGTAATACGACTCACTATAGGGAGA	201
		Reverse	TAATACGACTCACTATAGGGAGAATCGCTGCAATCTTCGTTGC	
**Se-vATPase**	qPCR	Forward	ACAGCTCAGACTCGCTGATG	268
		Reverse	AGAGAGTGTTCTTGGCGTCG	
**T7-Se-vATPase**	dsRNA	Forward	TAATACGACTCACTATAGGGAGAGTAGCCGCGGCCATACTATGT	348
		Reverse	TAATACGACTCACTATAGGGAGAGAATACAGAGTCTAGCTTTGG	
**Se-α tubulin**	qPCR	Forward	CGTGACGACGTGTCTGCGGT	167
		Reverse	GCGTGAGCTCGGGTACGGTG	
**T7-Se-α tubulin**	dsRNA	Forward	TAATACGACTCACTATAGGGAGACGTGACGACGTGTCTGCGGT	217
		Reverse	TAATACGACTCACTATAGGGAGAGCGTGAGCTCGGGTACGGTG	
**Px-vATPase**	qPCR	Forward	ACTCGATCGTGCTGGAGTTC	308
		Reverse	TTCGTCAGTGGAGAAGGTGC	
**T7-Px-vATPase**	dsRNA	Forward	TAATACGACTCACTATAGGGAGAGGCCATGTTCAGGAGCGA	418
		Reverse	TAATACGACTCACTATAGGGAGACGTCCTTGTCCTCCTGTTC	
	dsRNA	Forward	TAATACGACTCACTATAGGGAGAGTGCGACTACAACCCGAAGG	492

**T7-Px-Integrin β1**		Reverse	TAATACGACTCACTATAGGGAGAGGGGTAGAATGAGCCGAGTGC	
**Px-RL32**	qPCR	Forward	CCAATTTACCGCCCTACC	158
		Reverse	TACCCTGTTGTCAATACCTCT	
**Mp-vATPase**	qPCR	Forward	TTGCGAGTCATTCCTTCACCA	269
		Reverse	GACGGGAATCCCTTGGGTAA	
**Mp-EF1α**	qPCR	Forward	CTGATTGTGCCGTGCTTATTG	139
		Reverse	TATGGTGGTTCAGTAGAGTCC	
**Se-RL32**	qPCR	Forward	ATGCCCAACATTGGTTACGG	104
		Reverse	TTCGTTCTCCTGGCTGCGGA	
**T7-dsCON-GFP**	dsRNA	Forward	TAATACGACTCACTATAGGGAGACCGGTCCTCGAGATGGTGAGCAAGGGCG	520
		Reverse	TAATACGACTCACTATAGGGAGACGCGGGTACCTTACTTGTACAGCTCG	
